# Biopsychosocial factors associated with non-recovery after a minor transport-related injury: A systematic review

**DOI:** 10.1371/journal.pone.0198352

**Published:** 2018-06-12

**Authors:** Stella Samoborec, Rasa Ruseckaite, Darshini Ayton, Sue Evans

**Affiliations:** Department of Epidemiology and Preventive Medicine, School of Public Health and Preventive Medicine, Monash University, Victoria, Australia; Public Library of Science, UNITED KINGDOM

## Abstract

**Background:**

Globally, road transport accidents contribute significantly to mortality and burden of disability. Up to 50 million people suffer a transport-related non-fatal injury each year, which often leads to long-term disability. A substantial number of people with minor injuries struggle to recover and little is known about the factors leading to poor or non-recovery. The aim of this paper is to present a systematic review of biopsychosocial factors related to poor or non-recovery after a minor transport-related injury.

**Methods and findings:**

Studies were selected through searches of PubMed, Medline, Embase, and Cochrane library. Methodological quality was assessed using a Scottish Intercollegiate Guidelines Network (SIGN) critical appraisal checklist for quantitative cohort studies and Standards for Reporting Qualitative Research (SRQR) checklist for qualitative articles. Data were extracted using the Cochrane data extraction tool based on the biopsychosocial model of health (BPS). In total, there were 37 articles included. However, heterogeneity of the techniques and tools used to assess factors and outcomes across studies meant that pooling of results to determine biopsychosocial factors most predictive of poor or non-recovery was not possible. Hence, a narrative synthesis was conducted and shown multiple factors to be associated with poorer outcomes or non-recovery, most being identified in the biological and psychological domain of the BPS model. Factors that were the most representative across studies and have shown to have the strongest associations with poor or non-recovery were high initial pain intensity, pain duration and severity, pre-accident physical and mental health status and pain catastrophising.

**Conclusions:**

This review demonstrates the complexity of recovery and a challenge in reporting on predictors of recovery. It is evident that a range of multi-factorial biopsychosocial factors impact recovery. These factors are often inter-connected and multi-faceted and therefore, it was not feasible to select or focus on one single factor. In defining the most predictive factors, further research is required, yet the consensus around which tools to use to measure recovery outcomes is needed and is highly recommended. Regardless of the descriptive nature, the review demonstrated that high levels of post-injury pain are associated with poorer outcomes such as chronic pain and physical and mental disability. Therefore, early targeting of modifiable factors such as pain, pain catastrophizing and arising comorbidities such as PTSD, depression and anxiety may assist in reducing chronic pain and ongoing related disabilities.

**Systematic review trial registration number:**

Systematic review protocol was registered in International Prospective Register for Systematic Reviews (PROSPERO) on 14 December 2016. Registration number CRD42016052276.

## Introduction

Globally, injuries represent approximately 12% of the total burden of disease [1]. Specifically, road transport accidents are a major public health burden, affecting approximately 50 million people each year and representing a major public health, social and economic problem [2]. It is estimated that approximately 1.2 million people die each year as a result of transport accidents; the majority of whom are “vulnerable road users”–pedestrians and motorbike users [3]. The World Health Organisation (WHO) has estimated that injuries arising from the transport accidents will continue to rise and will be the third leading cause of disability by 2020 [3]. It has been shown that people involved in accidents have an 84% increased risk of developing chronic pain compared with the general population [4]. Consequently, post-accident chronic pain and related physical and mental disability have become a significant public health issue [5]. Further, it has been shown that people suffering chronic pain are in risk of developing mental health issues such as post-traumatic stress disorder (PTSD) and depression [6].

Injuries assessed as being minor in nature are the most frequently reported injuries following transport-related accidents [7]. However, there is no consistent and scientifically supported definition of minor injuries. In Canada, injury and compensation experts have defined a minor transport-related injury based on its recovery trajectory. Minor injuries include sprain, strain, Whiplash- Associated Disorders (WADs), contusion, abrasion, laceration or subluxation and any clinically associated sequelae [8]. The most commonly reported minor injuries following transport-related accidents are soft tissue injuries, including those related to muscles, tendons and ligaments. WADs are the most common soft tissue injuries and are present in up to 80% of minor transport injuries [9, 10]. Research has demonstrated that many chronic consequences arise from relatively minor injuries [11], and these may lead to more complex problems such as mental comorbidities, multiple hospitalisation and medical treatments and prolonged recovery.

In 1995, the Quebec Task Force (QTF) published findings following an extensive literature review of WADs and determined that it was not possible to report prognostic factors for recovery as there was a scarcity of appropriate prospective studies [12]. In 2001, Cote et al. identified that age, gender, baseline pain intensity and radicular signs/symptoms were significant predictors of recovery after whiplash injury [13]. In 2008 the Task Force on Neck Pain and Its Associated Disorders investigation of modifiable prognostic factors for neck pain recovery recommended that greater attention was required to investigate psychological factors impacting recovery [14]. Subsequent studies have demonstrated the importance of psychological factors [6], the role of medical providers and social insurers, and community and family support following a transport-related accident [15] in determining recovery outcomes following transport-related injuries. Therefore, recovery from transport-related accidents is clearly impacted by a range of different factors.

Given the above, the aim of this systematic review was to evaluate factors associated with non-recovery following minor injury, based on the biopsychosocial model of health [16]. This information will assist in identifying patients at high risk of poor recovery so that strategies can be implemented to reduce or prevent permanent or ongoing physical and mental disabilities.

## Materials and methods

### Search methods

An electronic search was conducted using the following databases: PubMed, Medline, Embase, and the Cochrane library. The initial search strategy was developed in Medline and then adapted to other databases. Search terms used to identify relevant studies included whiplash, contusion, abrasion, laceration, sprain and strain, joining with motor vehicle and transport accident. The search strategy is included in S1 File.

#### Inclusion and exclusion criteria

The systematic review included studies that registered patients following a minor transport-related injury and identified factors associated with poor or non-recovery. Articles were included if they satisfied the following criteria:

- Participants aged 18+
- Investigated patients who sustained a minor transport-related injury (based on an AIS score of 1 or the article’s description of minor injury)
- Assessed biological and/or psychological and/or social factors associated with non-recovery
- Defined the poor or non-recovery after a minor transport-related injury
- Used the biopsychosocial approach as a core model in identifying risk factors
- Used validated tools to measure health outcomes
- Reported a prognostic evaluation of the assessed factors
- Studies published in English
- Studies published between 2000 and December 2016

Studies were excluded if they did not use standardised and validated tools to assess a health outcome, and if they only used descriptive statistics (percentages %) to report associations between predictors and outcomes.

#### Study selection

During the first phase, articles were screened independently by two researchers on the foundation of title and abstract and were given an inclusion code (yes/no/unsure). The discrepancies on inclusion criteria were resolved by group discussions and consensus. Articles meeting the inclusion criteria underwent a full text review by two researchers to determine a final eligibility and confirm accuracy of the extracted data.

#### Protocol and registration

The review was conducted and reported in compliance with the Preferred Reporting Items for Systematic Review and Meta-Analyses (PRISMA- P). The protocol was registered in PROSPERO (International Prospective Register of Systematic Reviews) on December 14, 2016 and updated on September 22, 2017. A detailed description of the systematic review methodology is described in the published protocol [17].

#### Data extraction

Data from the relevant articles were assessed and extracted based on the Cochrane Data Extraction form [18].

Qualitative studies data were extracted into a separate table based on the Cochrane Qualitative Research Methods recommendations [19]. For qualitative studies, data extraction was a more iterative process. The themes identified in the studies that were relevant to the review question were extracted, regardless of whether or not they were illustrated directly by a quotation. This approach allowed data extraction to be more inclusive. The conceptual framework listed below was used as a guide for data extraction.

### Assessment of methodological quality

A quality appraisal for quantitative studies was assessed independently by two researchers using the Scottish Intercollegiate Guidelines Network (SIGN) criteria for assessing risk of bias in cohort studies [20]. Quality appraisal for qualitative studies was conducted by a principal researcher and a qualitative research expert. Standards for Reporting Qualitative Research (SRQR) were used to critically appraise the qualitative studies, as recommended by O’Brien and colleagues [21].

### Analysis

**Fig 1:**
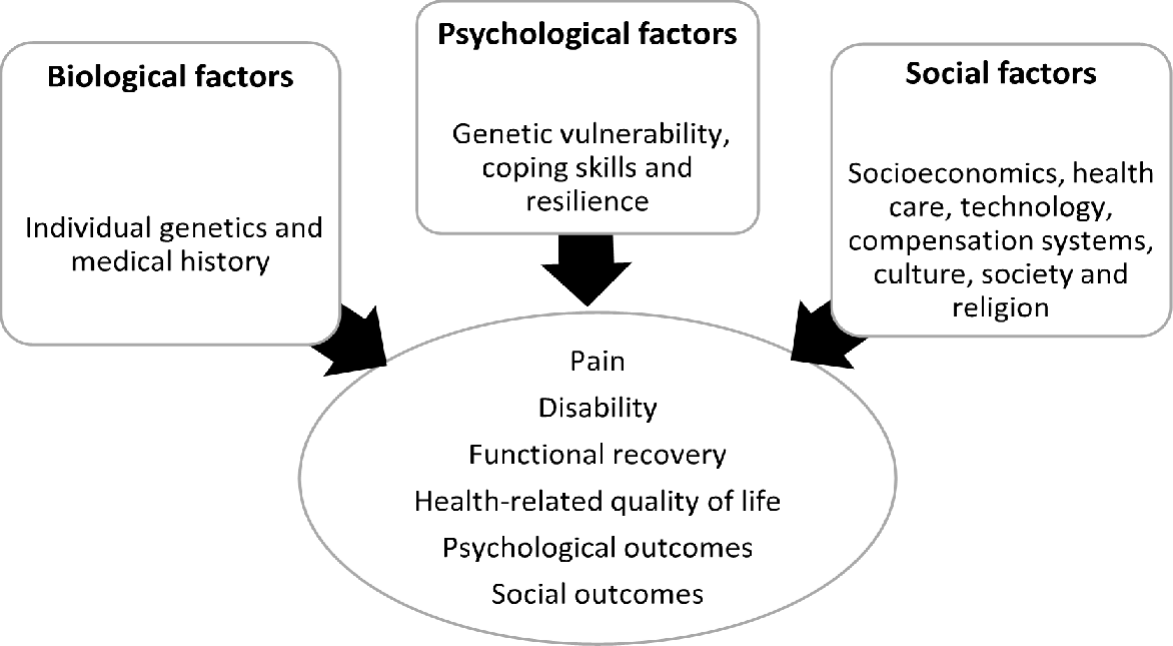
Conceptual framework for identifying biopsychosocial factors and health outcomes after a minor transport-related injury.

Poor recovery and non-recovery was defined according to the definitions provided in individual articles. There was heterogeneity of the population being studied; instruments, and definitions of prognostic factors being used, and outcomes measured. In addition, time intervals for the assessment of both prognostic variables and outcomes meant that no statistical pooling was performed for the analysis. As such, a narrative synthesis method was adopted following the general framework set out by Popay et al [22]. Narrative synthesis was used to summarise and explain the conclusions across the studies. The conceptual framework outlined in Fig 1 guided the analysis.

As per the published protocol, quantitative data were reviewed by two authors independently to classify factors associated with non-recovery into the biopsychosocial themes, using an inductive approach. We have included studies that have reported a significant association between factors and outcomes using p values, odds ratios, 95% confidence intervals, etc. The results were significant if a p value was less than 0.05. Univariate analysis were presented when multivariate results were not available, yet the confounding factors presented in multivariate analysis were highlighted in results.

Themes from the two qualitative studies were extracted and patterns and relationships were described independently by two authors. The results section is divided into thematic headings of prognostic factors related to non-recovery and themes identified as impacting recovery and quality of care.

## Results

### Selection of studies and study participants

**Fig 2:**
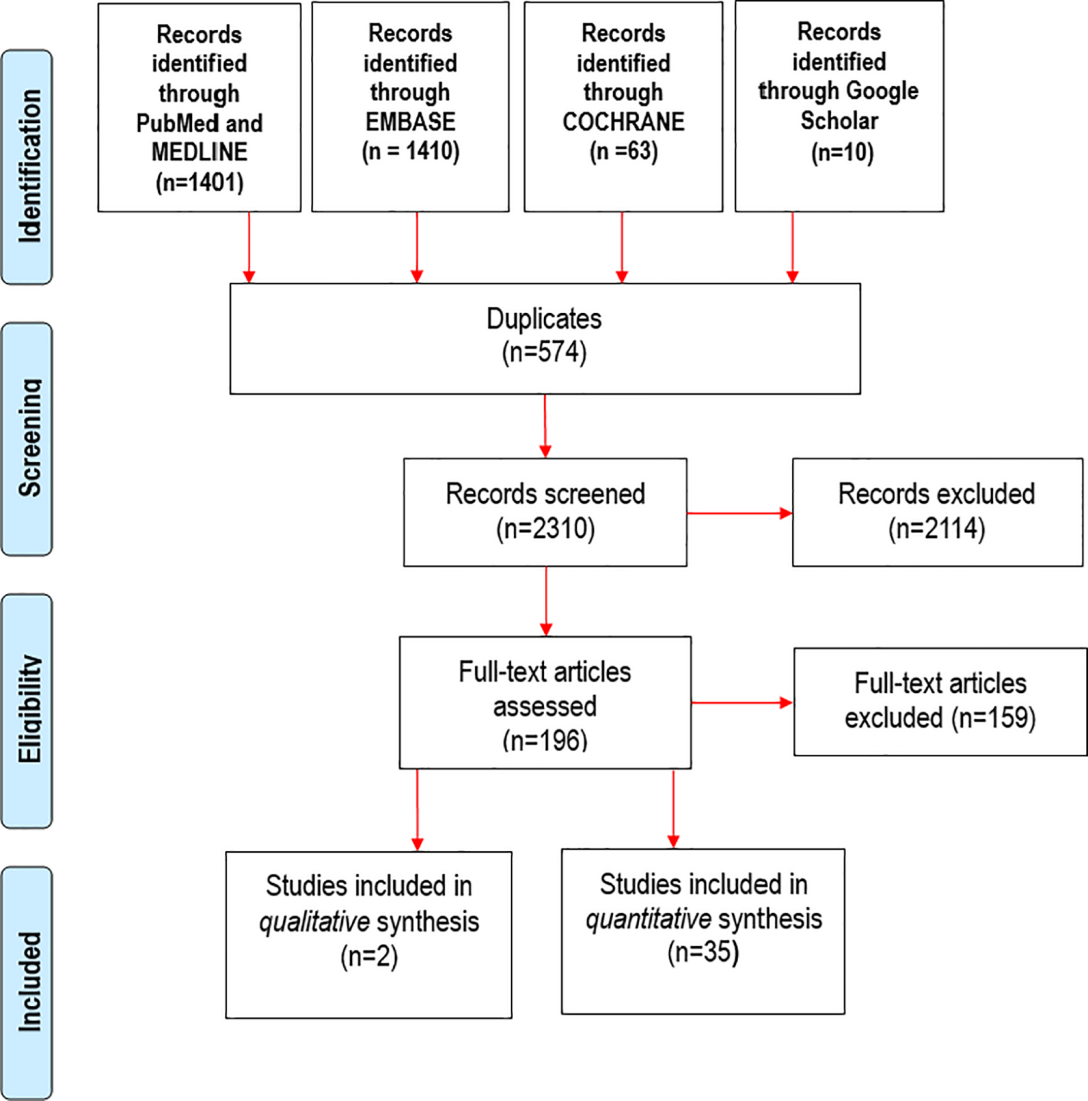
PRISMA flow chart of literature search.

The final search retrieved 2884 articles. After removing 574 duplicates, 2310 articles were screened for eligibility based on titles and abstracts with 196 selected for full text screening. Subsequently, 35 articles were included in the quantitative synthesis and 2 were selected for qualitative review (Fig 2). Of the 35 quantitative studies, 4 had a cross-sectional design and 31 had a cohort design (30 were prospective, 1 was retrospective).

The main characteristics of included studies are described in Table 1. Most investigated factors associated with non-recovery following whiplash injury (n = 22, 62%) followed by those studying other minor musculoskeletal injuries (n = 11, 32%). Two studies (6%) investigated mixed cohorts of those with minor and moderate injuries. The sample size varied from 22 to 6015 patients, with four studies enrolling less than 100 patients and four enrolling more than 3000 patients. As outlined in Table 1, most studies were conducted in Europe (n = 17, 50%), with nine (26%) conducted in Australia, seven (21%) in Canada and one (3%) in the US. Included participants were recruited from compensation schemes/insurance companies (n = 17), primary care practices and emergency departments (n = 10) and registries (n = 8). The follow up assessment ranged from 1 month to 24 months. Table 2 presents the results of the two qualitative studies included, one being conducted in Canada (involving 11 patients) and other in Australia (involving 32 patients).

**Table 1:** Main study attributes and findings from quantitative studies.

Author Year Country	Study type/ size	Study setting	Baseline	Follow up	Severity Type of injury	Factors identified	Outcomes assessed	Tools/ Measures	Association	Findings and implications for practice
**Bortsov et al [23]** **US** **2013**	Cohort 948	ED	6 weeks	6, 12 months	Minor musculoskeletal	Axial pain (pain in the neck, shoulders, and upper and lower back)	Pain intensity, distribution and interference	Regional Pain Scale (RPA) Brief Pain Inventory (BPI)	Significant association at 6 weeks, 6 and 12 months P values < 001	Widespread musculoskeletal pain has been shown to affect working ability, life satisfaction, and general health. Interventions seeking to achieve the greatest functional improvement in post-MVC pain should focus on axial pain or widespread pain.
**Bostick et al [24]** **Canada** **2012**	Cohort 72	Primary health care	6 weeks	3, 6 months	Minor musculoskeletal	Maladaptive beliefs (permanence and catastrophizing)	Pain severity Disability	Survey of Pain Attitudes (SOPA) Pain Beliefs and Perception Inventory (PBPI) Pain Catastrophizing Scale (PCS)	Significant association at 6 months follow up P values < 0.05	Pain beliefs represent potentially important predictors of outcome. The Medical Cure and Permanence subscales of the SOPA and PBPI are tools that could be used to measure these expectancy constructs.
**Buitenhuis et al [25]** **Netherlands** **2006**	Cohort 367	Insurance company	1 month	6, 12 months	Minor musculoskeletal	Fear of movement	Pain duration Anxiety	Tampa Scale of Kinesiophobia (TSK-DV)	Association before correction for early subjective neck symptoms P value < 0.001	This study showed that a relation exists between the score on the TSK-DV and the duration of neck symptoms.
**Buitenhuis et al [26]** **Netherlands** **2006**	Cohort 240	Insurance company	1 month	6, 12 months	Minor Whiplash	PTSD symptoms (avoidance, re-experiencing, and hyperarousal) Use of medication Back pain	Pain (severity and duration)	Self-Rating Scale for PTSD (SRS-PTSD)	Significant association at 1, 6 and 12 months follow up P values < 0.001	PTSD hyperarousal symptoms have a detrimental influence on the recovery and severity of whiplash complaints. NS
**Buitenhuis et al [27]** **Netherlands** **2008**	Cohort 140	Insurance company	1 month	6, 12 months	Minor Whiplash	Pain catastrophising Causal beliefs	Neck disability Neck pain	Neck Disability Index (NDI) Pain Catastrophizing Scale (PCS) Causal Beliefs Questionnaire Whiplash (CBQ-W)	Significant association at 6 and 12 months follow up P values < 001	Causal beliefs may play a major role in the perceived disability and course of neck complaints after motor vehicle accidents, whereas pain catastrophizing is predominantly related to concurrent disability.
**Carroll et al [28]** **Canada** **2006**	Cohort 6013	Insurance company	6 weeks	3, 6, 9, 12 months	Minor Whiplash	Pre-injury mental health problems	Post-injury depression	Centre for Epidemiological Studies Depression Scale (CES-D)		Pre-injury mental health problems increased the risk of later onset depressive symptoms and of a recurrent or persistent course of early onset depressive symptoms.
**Carroll et al [29]** **Canada** **2006**	Cohort 2290	Insurance company	6 weeks	3, 6, 9, 12 months	Minor Whiplash	Pain coping strategies (active and passive coping)	Self-perceived recovery Coping with pain	Pain Management Inventory (PMI)	Significant association at 6 weeks follow up P values <0.05	Even as early as 6 weeks post-injury, those who make frequent use of the types of coping strategies have a poorer prognosis for recovery. This highlights the clinical importance of early assessment of both the coping behaviours used and depressive symptomatology
**Carroll et al [10]** **Canada** **2009**	Cohort 6015	Insurance company	6 weeks	3, 6, 9, 12 months	Minor Whiplash	Recovery expectations	Self-perceived recovery Neck pain	Pain Disability Index (PDI)	Significant association at P values < 0.05	Those who expected to get better soon recovered over 3 times as quicker than those who did not expect to get better ever. Clinicians should assess expectations in order to identify those patients at risk of chronic whiplash, and future studies should focus on the effect of changing these early expectations
**Carroll et al [30]** **Canada** **2011**	Cohort 2986	Insurance company	6 weeks	4, 12 months	Minor Whiplash	Depression, anxiety, fear, anger, and frustration	Pain	Visual analogue scale—Pain	Significant association at 4 months follow up P values < 0.05	
**Carstensen et al [31]** **Denmark** **2008**	Cohort 740	ED and primary health care	3–10 days	12 months	Minor Whiplash	Pre-accident unspecified pain Female gender Pre-accident high psychological distress Low educational level Unemployment Blue collar worker Formal education>4 years	Pain RTW	Whiteley-7 (illness worrying) SCL-SOM (somatisation) SCL-OC (obsessive-compulsiveness) SCL-HOS (hostility) SCL-8 (mental illness) SCLDEP6 (depression) SCL-ANX4 (anxiety) Visual analogue scale (VAS)	Significant association at 12 months follow up P values < 0.05	Unspecified as opposed to specified pain (neck pain) before the collision is associated with poor recovery and high accumulation of pre-collision psychological distress is associated with considerable neck pain at follow-up. Together with socio-demographic vulnerability characteristics, these pre-collision elements give clinicians the possibility of detecting patients at high risk of a negative prognosis
**Carstensen et al [11]** **Denmark** **2015**	Cohort 719	ED and primary health care	3–10 days	12 months	Minor Whiplash	Weeks of sickness benefit Unemployment Social assistance	Negative change in provisional situation Pain	Visual analogue scale (VAS)	Significant association at 12 months follow up P values < 0.05	Sick leave before the collision strongly predicted prolonged recovery following whiplash trauma.
**Casey et al [32]** **Australia** **2010**	Cohort 246	Insurance company	3 months	NA	Minor Whiplash	Helplessness Pre-injury work status being affected	Pain Disability	Short Form 36 (SF36) Functional Rating Index (FRI) Pain Catastrophizing Scale (PCS)	Significant association p values < 0.001	The helplessness subscale of the PCS was the strongest predictor of disability and poorer health. Including additional information at claim notification, specifically the PCS and information on the effect the injury has on the working population could significantly improve claim screening processes identifying those in risk of non-recovery.
**Cobo et al [33]** **Spain** **2010**	Cohort 557	Primary health care	NS	6 months	Minor Whiplash	Old age Female gender Balance impairments Neck pain Self-employed workers	Pain Depression and anxiety Cervical functionality	Visual Analogue Scale (VAS) Goldberg Depression and Anxiety Scale (GDAS) Northwick Park Neck Pain Questionnaire (NPH)	Significant association at 6 months follow-up P values < 0.005	Factors that allow us to identify patients at risk for poor recovery are age, dizziness, and initial evaluation of neck pain. Proposing that initial evaluation to be made with validated, simple, and specific scales, such as VAS and NPH, for obtaining results at the same visit and, therefore, to start interdisciplinary actions at the beginning to improve results.
**Elbers et al [34]** **Netherlands** **2012**	Cohort 176	Insurance company	NS	NA	Minor Shoulder, arm, or hand Head or neck Hip, leg, or foot Trunk or back	Interaction with lawyers Interaction with social insurer	Quality of Life	European Quality of Life-5 Dimensions (EQ-5D)	Significant association at 12 months P values < 0.05	Interaction with lawyers was found to be fairer than with social insurers. This findings could implicate that it is possible to improve claimants’ health in compensation processes by enhancing procedural justice, e.g. by increasing the ability for claimants to express their views and feelings and by involving claimants in the decision-making process.
**Gopinath et al [35]** **Australia** **2014**	Cohort 364	Injury Registry	NS	12, 24 months	Minor musculoskeletal	Pain severity Pain catastrophizing	Chronic pain	Numeric rating scale (NRS) Pain- Related Self-Statements Scale-Catastrophizing Orebro Musculoskeletal Pain Screening Questionnaire (OMPSQ	Significant association at 12 and 24 months follow up p values < 0.001	This results could give clinicians a better understanding and possibility of detecting patients at high risk of a negative prognosis after the collision.
**Gopinath et al [36]** **Australia** **2015**	Cohort 364	Injury Registry	25–92 days	12, 24 months	Minor musculoskeletal	Hospitalisation (>24hours < 7 days)	Quality of life Pain	European Quality of Life-5 Dimensions (EQ-5D) SF12 PCS and MCS Pain numeric rating scale score (NRS)	Significant association at 12 months P values < 0.05	Being hospitalised following a non-catastrophic injury sustained in a road transport crash independently predicts poorer physical health or functioning 12 months later, but not at 24 months.
**Gopinath et al [37]** **Australia** **2015**	Cohort 364	Injury Registry	25–92 days	12, 24 months	Minor musculoskeletal	Older age (<65)	Quality of life Pain	European Quality of Life-5 Dimensions (EQ-5D) SF12 PCS and MCS Pain numeric rating scale score (NRS)	Significant association at 24 months follow up p values < 0.05	Older compared to younger participants who sustained a mild/moderate injury following a road-transport crash demonstrated poorer physical functioning and general health at 24 months. This findings will highlight the need for interventions targeting enhanced recognition and management of poorer physical functioning and overall health status in older compared to younger
**Gopinath et al [38]** **Australia** **2015**	Cohort 364	Injury Registry	NS	12, 24 months	Minor musculoskeletal	Pre-injury health status Pre-injury chronic condition Older age > 45	RTW Pain Return to usual activities Quality of life	European Quality of Life-5 Dimensions (EQ-5D) Numeric rating scale (NRS) SF 12 PCS and MCS Orebro Musculoskeletal Pain Screening Questionnaire (OMPSQ)	Significant association at 24 months P values < 0.05	Higher SF-12 physical component summary (PCS), and EQ-5D visual analogue scale (VAS) scores were mutually independent predictors of returning to usual activities 24 months later. These determinants could be measured early in the recovery process and be potentially amenable to intervention.
**Holm et al [39]** **Sweden** **2007**	Cohort 1187	Insurance company	6 weeks	6 months	Minor Whiplash	Pre-accident neck pain Pre-accident headache Pre-accident poor general health Low educational level Living alone	Pain intensity	Visual Analogue Scale (VAS) SF36	Significant association at 6 months P values < 0.05	Both biomedical and psychosocial factors are associated with initial neck pain intensity. This study suggest that the case history should include socioeconomic status and detailed prior health state, because these may be of importance for understanding differences in pain and course of the injury.
**Holm et al [40]** **Sweden** **2008**	Cohort 1032	Insurance company	23 days	6 months	Minor Whiplash	Self-reported expectation of recovery	Self-perceived disability	Numerical rating scale—recovery (NRS) Pain Disability Index (PDI)	Significant association at 6 months P values < 00.05	Individuals’ expectations for recovery are important in prognosis, even after controlling for symptom severity. Early assessment of expectations for recovery to be made, in order to identify people at risk for poor prognosis after WAD.
**Holm et al [41]** **Sweden** **2006**	Cohort 266	Insurance company	6 weeks	4, 6, 12 months	Minor Whiplash	Pre-accident depressive symptoms Neck pain intensity Reporting >3 pain-associated symptoms Reporting 4 or 5 painful body areas	Widespread pain Post-injury depression	Centre for Epidemiological Studies Depression Scale (CES-D) Visual analogue scale (VAS)	Signifiant association P values < 0.05	Subjects with WAD who report early depressive symptoms and more severe neck injury symptoms are at risk of developing WP after MVC.
**Hours et al [42]** **France** **2013**	Cohort 380	Road crash trauma registry	Ns	12 months	Minor musculoskeletal	Age (> 35) Female gender Educational level Pre-accident financial problems Pre-accident mental history	QOL	World Health Organization Quality of Life tool (WHOQOL-BREF)	Significant association at 12 months follow up p values < 0.05	Sociodemographic factors, pre-accident psychological history prior to the accident, and PTSD were the main factors influencing QOL, rather than whether the injury was whiplash. This results should be useful in attracting the attention of both clinicians and the public administration to patients at risk of suffering from consequences after a mild accident.
**Kenardy et al [43]** **Australia** **2015**	Cohort 382	Insurance company	NS	6, 12, 24 months	Minor musculoskeletal	Pain intensity Pre-accident mental health history PTSD Anxiety Depression Perceived threat to life Older age Low expectation of RTW	Self-reported disability	WHO-DAS-II Composite International Diagnostic Interview module for PTSD Generalized anxiety disorder (GAD) Alcohol Use Disorder Identification Test Orebro Musculoskeletal Pain Questionnaire Multidimensional Scale of Perceived Social Support	Significant association at 6, 12 and 24 months follow up p values < 0.05	Claimants with predominantly minor physical injuries report high disability, particularly when comorbid psychiatric disorders are present, pain is high, and expectations regarding return to work are low. Developing tools for detecting those at risk of poor recovery after an RTC is necessary for informing policy and practice in injury management and post-injury rehabilitation
**Kongsted et al [44]** **Denmark** **2007**	Cohort 737	ED Primary health care	NS	12 months	Minor Whiplash	Initial pain intensity Acute stress reaction	Pain Disability General health RTW	SF-36 Copenhagen Neck Functional Disability Scale Impact Event Scale (IES)	Association at 12 months follow up P values < 0.05	The acute stress response was significantly related to the development of chronic WAD, particularly clear in those with low baseline pain.
**Littleton et al [45]** **Australia** **2010**	Cohort 95	Accident Care Evaluation Study	Median 8 days	6, 12 months	Minor/moderate	Claiming compensation	Physical recovery Mental health Functional recovery	SF-36 PCS and MCS Hospital Anxiety and Depression Scale (HADS) Functional Rating Index (FRI)	Significant association at 12 months follow up p values < 0.05	Claiming compensation and psychological factors were independent predictors of worse health status at 12 months.
**Littleton et al [46]** **Australia** **2012**	Cohort 193	ED	4 weeks	NA	Minor/moderate	Being at fault	Physical recovery Mental health Functional recovery	SF-36 PCS and MCS Hospital Anxiety and Depression Scale (HADS) Functional Rating Index (FRI)	Significant association P values < 0.05	The ‘not at fault’ (NAF) group demonstrated more emotional and mental disturbance than the ‘at fault’ (AF) group; and this was significantly worse for females. Treatment strategies should focus on addressing early pain and disability as well as providing appropriate psychological interventions, particularly for people not at fault following RTC.
**Myrtveit et al [47]** **Norway** **2015**	Cohort 740	Primary care	10 days	12 months	Minor Whiplash	Preferring medications Sickness absence Taking medications Passive coping skills and preferences	Neck pain RTW	Visual analogue scale (VAS) Vanderbilt Pain Management Inventory (VPMI)	Significant association at 12 months follow up P values < 0.05	Coping preferences are associated with the development of chronic neck pain and reduced capability to work following whiplash trauma. This study suggest that attention towards acute whiplash patients’ coping preferences can lead to identification of individuals at high risk of poor recovery
**Nieto et al [48]** **Spain** **2013**	Cohort 147	Primary care	3 months	6 months	Minor Whiplash	Initial pain intensity Initial disability Fear of movement	Pain intensity Disability	Pain Catastrophizing Scale (PCS) Tampa Scale of Kinesiophobia (TSK) Numerical rating scale (NRS-11) Neck Disability Index (NDI)	Significant association at 6 months follow up P values < 0.05	Biopsychosocial model is a proper template to design and implement programs to prevent long-term pain and disability in WAD. Pain intensity, disability, and fear of movement should be attended to and reduced as soon as possible following a whiplash injury.
**Nijs et al [49]** **Belgium** **2011**	Cohort 143	ED	NS	10, 26 months	Minor Whiplash	Social support (inadequacy, self-satisfaction and resentment)	Physical health Mental health QOL	Neck Disability Index (NDI) SF-12 Anamnestic Comparative Self-Assessment (ACSA) Dutch Personality Questionnaire Social Support List	Significant association P values < 0.001	The present study suggests that social support and personality traits (i.e. inadequacy, self-satisfaction and resentment) are associates of long-term functioning following whiplash injury.
**Ozegovic et al [50]** **Canada** **2010**	Cohort 2335	Insurance company	Within 42 days post-accident	NA	Minor Whiplash	Depressive symptomatology Age > 50 High initial pain intensity and duration Lower educational level Low income	RTW expectations	Numerical analogue scale (NAS) Centre for Epidemiologic Studies Depression Scale (CES-D) SF-36	Significant association P values < 0.05	Depressive symptomatology, and greater initial pain (greater percentage of body in pain and greater intensity of neck pain) were strongly associated with lower return-to-work expectation. These results support using a biopsychosocial approach to evaluate expectancies and their influence on important health outcomes.
**Ozegovic et al [51]** **Canada** **2010**	Cohort 6015	Insurance company	Within 42 days post-accident	NA	Minor Whiplash	Initial neck pain intensity Depressive symptomatology	Recovery expectations	Numerical analogue scale (NAS) Centre for Epidemiologic Studies Depression Scale (CES-D) SF-36	Significant association P values < 0.05	Two of the strongest associated factors with non-recovery were depressive symptomatology and initial neck pain intensity. Further research is needed to assess the impact of an individuals’ prior understanding of an event on expectations; the potential of time varying components of expectation over the recovery process.
**Phillips et al [52]** **Canada** **2009**	Cohort 3452	Insurance company	11 days post-accident	6 weeks, 3, 6, 9, 12 months	Minor Whiplash	Older age > 50 Initial neck and low back pain Dizziness Vision and hearing problems Numbness/tingling in arms/hands Anxiety Prior mental health problems Poorer general health	Post-injury depression	Centre for Epidemiological Studies Depression Scale (CES-D) Numerical rating scale (NRS-11)	Significant association P values < 0.05	Predictors of persistent depression included older age, greater initial neck and low back pain, post-crash dizziness, vision and hearing problems, numbness/tingling in arms/hands, anxiety, prior mental health problems, and poorer general health. Recognition of these underlying risk factors may assist health care providers to predict the course of psychological reactions and to provide effective interventions.
**Takasaki et al [53]** **Australia** **2013**	Cohort 40	NS	NA	3, 72 months	Minor Whiplash	Cognitive impairments Physical impairments	Self-reported driving difficulty	Angular velocity of head rotation (MV) Head rotation range of motion (ROM) Dizziness Handicap Inventory short form Neck Pain Driving Index (NPDI) Neck Disability Index (NDI)	Significant association P values < 0.001	Physical and cognitive impairments independently contributed to self-reported driving difficulty in chronic WAD beyond neck pain, dizziness, and symptom duration. This study provides clinicians with useful suggestions to direct management of patients with chronic WAD who report driving difficulty. Clinicians should not only address symptoms such as neck pain and dizziness as well as motor and sensorimotor functions, but also cognitive impairments.
**Thompson et al [54]** **UK** **2010**	Cohort 55	Primary health care	NS	NA	Minor Whiplash	Pain catastrophizing Lower functional self-efficacy beliefs	Pain Disability	Neck Disability Index (NDI) Pain Catastrophising Scale (PCS) Tampa Scale for Kinesiophobia (TSK) Pain Vigilance and Awareness Questionnaire (PVAQ)	Significant association P values < 0.05	Interventions which aim to reduce catastrophizing and enhance functional self-efficacy beliefs should be included alongside conventional physiotherapy interventions when treating patients with chronic whiplash-associated disorder.
**Wynne-Jones et al [55]** **UK** **2006**	Cohort 957	Insurance company	1 month	12 months	Minor musculoskeletal	Older age Injury severity Pre-accident health-seeking behaviour Pre-accident somatisation	Widespread pain	General Health Questionnaire (GHQ) Illness Attitudes Scale (IAS) Somatic Symptoms Checklist	Significant association at 12 months follow up P values < 0.05	This study identified factors that independently predict the onset of WP following a motor vehicle collision. Early identification of this “at-risk” group may allow the targeting of preventive management. in those at highest risk of developing future symptoms

**Table 2:** Qualitative studies attributes and themes assessed.

Study	Aim	Research question	Study design	Data collection methods	Sample characteristics	Context and setting	Approaches to data analyses and interpretation	Factors/ themes selected
**Murgatroyd et al [56]** **Australia** **2015** The perceptions and experiences of people injured in motor vehicle crashes in a compensation scheme setting: a qual study	To explore/identify how to positively influence distinct compensation systems so that people’s interactions with insurers lead to improved rather than poorer health post injury; whilst maintaining scheme equity and affordability.	1. What are people’s perceptions and experiences of the claims process after sustaining a compensable injury in a motor vehicle crash? 2. Why do people seek legal representation? 3. How can people be assisted following a compensable injury and their experience with the claims process improved?	Exploratory study	Socio-demographic and injury data obtained from cohort study database. Five focus groups with 32 attendees.	32 participant Age range: 22–79 Mean age: 47 12 males 20 females 72% post high school qualifications. 38% working as managers/professionals. 19 had returned to work at time of FG. 12 had their claim settled at time of FG. 12 legally represented.	New South Wales Australia Motor Accidents Authority–government insurance compulsory third party personal insurance scheme. NSW– 7 million pop. 26753 road causalities in 2010/2011 (killed or injured). 39% made claims	Not actually grounded theory! Thematic analysis–open coding Coded independently by 2 researchers and triangulation of themes by a 3rd researcher	**Primary themes:** 1. Complexity of the claims process 2. Requirements of legal representation 3. Injury recovery expectations 4. Importance of timely healthcare decision making 5.Improvements for injury recovery 6. Desire for financial compensation 7. Lack of trust by insurers 8. Medico-legal assessments 9. Family and social support
**Lindsay et al [57]** **Canada** **2016** Patients’ experiences with vehicle collision to inform the development of clinical practice guidelines: a narrative inquiry.	To explore the experiences and describe the recommendations of injured persons to inform the development of a new evidence-informed CPG for the management of common transport injuries in Ontario, Canada.	1. What is the injured persons’ experience with health care following transport collision-caused injury? 2. What would injured persons want a group of experts (healthcare professionals, scientists, insurers, and public representatives, policy makers) to know about their experience as they make decisions about the development of guidelines for the management of minor injuries after collisions?	Narrative inquiry	11 participants Participated in two interviews within 2 weeks of each other. First interview 1 hour face to face. 2nd interview 30 minutes–completed by phone	11 participants 8 injured in cares, 2 pedestrians injured by car, 1 injured in motor cycle collision. 5 men aged 35–73 6 women aged 39–67 7 employed, 2 retired, 1 unemployed 1 student Injuries: whiplash, contusion, headaches, swollen joints, bruising on torso,	Greater Toronto Area including Niagara, Kingston, and Sudbury. Minor Injury Guidelines developed by the Ontario Protocol for Transport Injury Management	Narrative analysis Identification of narrative plotlines.	1. Importance of terminology 2. Partnerships and shared decision making 3. Impact of emotional distress 4. Understanding the system

### Summary of methodological assessment of individual studies

This methodological assessment focused largely on the risk of bias in individual studies and followed step by step quality guidance provided by the SIGN. The SIGN criteria that always applies to cohort studies has been applied as majority of studies were of a prospective cohort design. The overall quality score ranged from 1 to 6 based on the SIGN questionnaire and quality indicator was assigned. The results of methodological quality are presented in Table 3 and show that studies were mostly of moderate to high quality. Majority of the studies had clearly identified questions and outcomes and used reliable assessment of exposure. Twelve studies were of a highest quality (34%), fifteen of moderate quality (44%) and 8 studies of a low quality (22%). As per a study design nature, where no studies have studied more than one group, no studies were able to blind the outcome to the exposure status, yet, this has not affected the quality results. However, 18 studies (52%) did not identify potential confounders and 14 (40%) did not assess prognostic factors at more than one-time point. The findings appear to be reasonably consistent across the range of study populations and study designs, yet, the heterogeneity present in tools used to measure factors and outcomes meant that no statistical pooling was feasible.

**Table 3:** Quality appraisal of the quantitative studies.

**Study**	**Appropriate and clearly focused question**	**The outcomes are clearly defined**	**Method of assessment of exposure is reliable**	**Prognostic factor is assessed more than once**	**Potential confounders are identified**	**Confidence intervals are provided**	**Quality indicator**
Bortsov et al	+	+	+	+	?	+	++
Bostick et al	+	+	+	+	?	+	++
Buitehuis et al 2006	+	+	+	+	?	+	++
Buitehuis et al 2006	+	?	+	+	?	+	+
Buitehuis et al 2008	+	+	+	+	?	+	++
Carroll et al 2006	+	+	+	+	+	+	+++
Carroll et al 2006	+	+	+	+	-	+	++
**Study**	**Appropriate and clearly focused question**	**The outcomes are clearly defined**	**Method of assessment of exposure is reliable**	**Prognostic factor is assessed more than once**	**Potential confounders are identified**	**Confidence intervals are provided**	**Quality indicator**
Carroll et al 2009	+	+	+	-	+	+	++
Carroll et al 2011	+	+	+	+	+	+	+++
Carstensen et al 2008	+	+	+	-	+	+	++
Carstensen et al 2015	+	+	+	+	+	+	+++
Casey et al 2011	+	+	+	-	-	+	+
Cobo et al	+	+	+	-	?	+	+
Elbers et al	+	+	+	-	+	+	++
Gopinath et al 2014	+	+	+	+	+	+	+++
Gopinath et al 2015	+	+	+	+	+	+	+++
Gopinath et al 2015	+	+	+	+	+	+	+++
Gopinath et al 2015	+	+	+	+	+	+	+++
Holm et al 2007	+	+	+	+	+	+	+++
Holm et al 2007	+	+	+	-	+	+	++
Holm et al 2008	+	+	+	+	+	+	+++
Hours et al 2014	+	+	+	+	?	+	++
Kenardy et al	+	+	+	+	+	+	+++
Kongsted et al	+	+	+	+	?	+	++
Littleton et al 2011	+	+	+	-	+	+	++
Littleton et al 2010	+	+	+	+	?	+	++
Myrtveit et al	+	+	+	+	?	+	++
Nieto et al	+	+	+	+	+	+	++
Nijs et al	+	+	+	-	-	+	+
Ozegovic et al 2010	+	+	+	-	-	+	+
Ozegovic et al 2010	+	+	+	-	-	+	+
Phillips et al	+	+	+	+	+	+	+++
**Study**	**Appropriate and clearly focused question**	**The outcomes are clearly defined**	**Method of assessment of exposure is reliable**	**Prognostic factor is assessed more than once**	**Potential confounders are identified**	**Confidence intervals are provided**	**Quality indicator**
Takasaki et al	+	+	+	-	-	+	+
Thompson et al	+	+	+	-	-	+	+
Wynne-Jones et al	+	+	+	+	?	+	++

**+** meeting criteria;—not meeting criteria;? not stated

**High quality (+++):** Little or no risk of bias (6/6)

**Acceptable (++):** Most criteria met (5/6)

**Low quality (+):** Most criteria not met, or significant flaws relating to key aspects of study design and methodology (≤4/6)

Results of the methodological assessment of the two qualitative studies included are presented in Table 4 and both were considered to be of a high quality.

**Table 4:** Qualitative literature appraisal SRQR.

Criteria	Study 1	Study 2
Title	-	+
Abstract	+	+
Problem formulation	+	+
Purpose or research question	+	+
Qualitative approach and research paradigm	+	+
Researcher characteristics and reflexivity	-	+
Context	+	+
Sampling strategy	+	+
Ethical issues pertaining to human subjects	+	+
Data collection methods	+	+
Data collection instruments and technologies	+	+
Units of study	-	-
Data processing	+	+
Data analysis	+	+
Techniques to enhance trustworthiness	+	+
Synthesis and interpretation	+	+
Links to empirical data	+	-
Integration with prior work, implications, transferability, and contributions to the field	+	+
Limitations	+	+
Conflicts of interest	+	+
Funding	+	+

**+** meeting criteria;—not meeting criteria;? not stated

### Outcome measures and instruments used across studies

The definition and outcome measures for recovery varied across studies. Recovery was defined variably in terms of morbidity (e.g. degree of recovery from pain, functional disability and mental disorder), return to pre-injury health status and return to work. In total 12 standardised instruments were used across the studies to measure pain; nine using a Visual Analogue Scale and eight using a Pain Numerical Rating Scale. Disability was assessed with six different tools; the most commonly used instrument being the Neck Disability Index used in 8 studies. The Pain Catastrophizing Scale and Short Form 36 were used in 6 studies to assess pain while the Centre for Epidemiological Studies Depression Scale and Hospital Anxiety and Depression Scale were the most commonly used instruments to assess depression and anxiety. Even though the majority of selected studies were of prospective cohort design, the large number of tools used to measure recovery outcomes meant that it was not possible to statistically assess the main predictors of recovery. This obviously raises an issue and it is an area requiring further research as currently there is no consensuses amongst researchers around what is the most appropriate tool to use to measure recovery outcomes after transport-related injuries.

Even though this review was not able to conduct the statistical analysis the narrative framework was adopted and descriptive results are presented below.

### Narrative synthesis

A total of 37 articles were included in the narrative synthesis. After identifying common biopsychosocial factors among the variables from the quantitative studies and extracting themes from the qualitative studies, fourteen relevant factors were described across the BPS model of health domains. The data were extracted following the conceptual framework described in Fig 1. These factors/themes are outlined in Table 5 and described in detail below:

**Table 5:** Factors impacting recovery as identified in the literature.

Biological domain
**Pain (types, intensity and duration)**
**Age**
**Sex**
**Pre–accident physical or mental disability/chronic condition**
Psychological domain
**Pain catastrophizing and causal beliefs**
**Recovery expectations and coping skills**
**PTSD, anxiety and depression**
**Pre-accident health-seeking behaviour and somatisation**
Social domain
**Previous unemployment and low educational level**
**Hospitalisation status**
**Procedural justice and compensation process**
**Lack of trust by insurers**
**Importance of timely healthcare decision making**
**Family and social support**

### 1. Biological domain

#### 1.1. Pain-related factors

In this review, pain was identified as a predictor of recovery and also as a measure of recovery. In terms of being a predictor of recovery, different types, intensity and duration of pain were described across the studies. The type of pain described included widespread (pain in the neck, shoulders, and upper and lower back), unspecified, neck, mid and low back pain and headache with frequently reported symptoms such as numbness/tingling in arms/hands, pins and needles sensations, and dizziness.

Widespread pain was found to be related with working incapacity, poor quality of life and poor general health. The study modified results for overall pain intensity and found that overall pain has the greatest impact on six of the seven life functions measured at six and twelve months follow up [23]. Mid-back and lower-back pain, frequently reported by women, were related with poor expectations of recovery. Results from the largest cohort included in this review (n = 6015) shown that the presence of headache, low back pain, greater percentage of body in pain, greater initial pain, and greater intensity of neck pain were associated with low recovery expectations, which lead to poor recovery outcomes [51]. High initial pain intensity and pain duration were associated with poor recovery expectations [50], low expectations of return to work [50], chronic depression [52], and self-reported driving difficulty [53].

In terms of being an outcome measure, pain severity, chronic pain and widespread pain were measured in multiple studies and revealed that pain catastrophizing, pain-related work disability, expectancy beliefs [24], acute stress response [44], pain-related emotions [30], depressive symptomatology [39], initial pain intensity [11, 39], sickness absence or health seeking behaviour and being referred to physiotherapist/chiropractors [47] were important predictors of pain-related outcomes. Furthermore, initial pain intensity and pain duration were significant predictors of pain severity in patients with whiplash at 6 months in a cohort study (n = 123) [48]. Widespread pain was associated with three or more pain-associated symptoms, initial high levels of pain, and depressive symptomatology [41] [43].

#### 1.2. Age

Systematic reviews published in 2001 [13] and 2003 [58] have reported conflicting findings in relation to the association of age and relevant outcomes following whiplash. Further to these two studies, an additional four studies published after 2003 investigated the relationship between age and recovery after minor transport-related injury. These studies all demonstrated that increasing age was associated with poorer health outcomes. A prospective cohort study (n = 252) investigating minor transport injuries found that patients aged ≥ 65 years demonstrated poorer physical functioning and general health at 12 and 24 months post-injury compared to those aged <65 years. [59] Two studies investigating recovery after whiplash confirmed that age was a predictor of non-recovery [33] and persistence of pain [23]. In studies investigating the effect of mental health on long term disability, older age has predicted higher disability, but only in patients suffering post-traumatic stress disorder (PTSD). A large study (n = 5211) investigating depressive symptomatology in whiplash patients also reported that those 40 years of age and older were 60% more likely than those aged under 40 years to develop depressive symptomatology in the first 6 weeks post-accident [28].

#### 1.3. Sex

Sex was found to be associated with poor recovery in four studies. Specifically, in a study of 740 patients from the emergency department and primary health care female sex was associated with poorer return to work at 12 month follow up [31]. Another study reported that female sex was associated with poor general health 6 months post-injury and was identified as prognostic factor of poor recovery [33]. On the other hand, one cohort study, which was the largest study included (n = 6015) reported that male sex was associated with poor expectations of recovery, yet the reported association was not strong (OR = 1.22) in comparison to a female sex (OR = 1.0) [51]. A cross-sectional study involving 2335 whiplash patients revealed that male sex was associated with lower return to work expectations [50]. The conflicting evidence on the association between sex and recovery suggests that it might not have a direct association with poorer outcomes but may potentially reflect more complex interactions between prognostic factors.

#### 1.4. Pre–accident physical and mental health status

The significance of personal health characteristics before the accident, such as chronic pain, depressive symptomatology and cognitive impairments as potential predictors of recovery were highlighted in seven studies. A study from Denmark (n = 740) found that pre-accident pain was predictive of return to work outcomes at 12 month follow up and suggested that pre-accident factors are crucial when measuring recovery after transport injury [31]. An Australian study evaluating social outcomes following minor injury in 364 patients found that pre-injury general health status and presence of chronic conditions were independent predictors of returning to usual activities [36]. It has also been demonstrated that increased pre-accident psychological distress is associated with non-recovery (28) and pre-accident mental health problems increase the post-trauma risk of depressive symptoms and adversely affect patients’ quality of life regardless of the type of minor injury sustained [28, 42]. Another Australian study reported a significant association between pre-accident mental health conditions and mental disability post-accident [43]. A prospective study that investigated a range of biopsychosocial factors found that having a pre-accident chronic condition was associated with delay in returning to work at 24 months [36].

### 2. Psychological domain

#### 2.1. Pain catastrophising and causal beliefs

A cross-sectional study involving a smaller cohort (n = 55) recruited from a physiotherapy department reported that patients with a higher level of catastrophising was related to increased reporting of pain intensity [54]. However, the results were drawn from a univariate analysis and multivariate did not show any significant association between these abovementioned cognitive factors and pain intensity. Another small prospective cohort study (n = 72) reported that expectancy beliefs were negatively associated with pain intensity at 6 month follow up in patients with whiplash, and that catastrophising was predictive of increased pain intensity at 3 and 6 months post-injury [24]. A larger prospective study (n = 140) revealed in their multiple regression model that pain catastrophising and causal beliefs were associated with pain severity at 6 and 12 months follow up [25]. An Australian study (n = 252), which recruited their participants through a compensation database confirmed these results in their multivariable analysis and demonstrated that pain catastrophising was associated with pain disability in a minor injury cohort at 12 and 24 months [35].

#### 2.2. Recovery expectations and coping skills

Recovery expectations have been identified as an important factor in predicting patient’s recovery in two studies. A large population based study (n = 6015) investigated the association between self-reported expectations of recovery and self-reported global recovery, including pain severity and pain related emotions, and demonstrated that those who expected to recover soon, recovered three times as quickly as those who expected they would never get better [10]. Another large cohort (n = 1023) from Sweden demonstrated that individuals who stated they were less likely to make full recovery at 6 month follow up were more likely to have higher disability compared to participants who stated they were likely to make full recovery. Moreover, after controlling for symptom severity, a negative association was also found among individuals with moderate disability [40].

Conversely, qualitative studies that explored patients’ recovery expectations found that patients’ expectations were influenced by previous experiences with social and healthcare professionals and their current beliefs of their injury and recovery. Therefore, this study suggested that negative experiences led to poorer recovery expectations as patients who reported minimal pain and disability observed their recovery as being easier when compared with patients who reported higher levels of pain [56].

Poor coping skills seemed to have a negative influence on outcomes; especially in terms of how patients deal with pain after transport injury. For example, a study that assessed the relationship between pain coping strategies and recovery found that even as early as six weeks post-injury, those with passive coping strategies and skills had poorer recovery outcomes; and that this was exacerbated when supplemented by early post-injury depressive symptomatology [29].

#### 2.3. PTSD, anxiety and depression

Depressive symptomatology such as PTSD, anxiety and depression have been reported as predictors as well as outcomes in patients with minor injuries following a transport accident. PTSD was found to be associated with poor quality of life [42], disability [43] and severity of pain [41]. A study involving 240 participants who made a compensation claim for soft tissue injury in Netherlands revealed that the initial number of hyperarousal symptoms were found to be predictive of poorer health at 6 and 12 months in a multivariate analysis [27]. Also, pain related emotions such as frustration, anger and anxiety were found to be strongly related to poor recovery, especially in patients who were not deemed at fault for the accident [30]. Depressive symptoms were also found to be associated with development of widespread pain in a large prospective cohort study [41].

In terms of being an outcome, chronic depressive symptomatology was found to be associated with presence and severity of whiplash symptoms such as pain [27], passive coping strategies [29], involvement in the compensation process [45], older age, greater initial neck and low back pain, and prior mental health problems [52]. Furthermore, a large cohort study involving 5211 whiplash participants who reported no previous mental health issues demonstrated that 42.3% developed depressive symptomatology within 6 weeks of their injury. However no regression analyses were conducted to test the predictive validity of other factors such as anxiety and PTSD [28].

#### 2.4. Pre-accident health-seeking behaviour

Prognostic capacity of pre-accident health seeking behaviour has not been evaluated in sufficient studies to allow conclusions on the prediction capability to be drawn. However, two studies have investigated whether these factors were associated with recovery. One large prospective study (n = 719) found that people with sickness behaviour who took sick leave for more than 12 weeks in the last 5 years reported considerable neck pain at 12 months follow up [11], while the other study reported that pre-accident sickness behaviour, being on medications and sickness absence were associated with poor recovery, described as reduced work capacity and chronic pain [47].

### 3. Social domain

#### 3.1. Employment and educational level

Studies have shown that being previously unemployed, having pre-accident work status affected post–injury [11, 32] and having a low education level [50, 51] were barriers to returning to work after transport-related accidents. In a prospective cohort study involving 740 patients recruited from emergency departments and primary health services, a multivariate analysis revealed that unemployment, low educational level and being a blue collar worker were associated with poorer work capacity 12 months post-injury [31].

#### 3.2. Hospitalisation status

Two prospective cohort studies have identified an association between hospital stay and poor recovery. One study involving 246 participants with minor transport-related injuries revealed that not being admitted to hospital was associated with a 44% higher likelihood of returning to work after 24 months. Another study used compensation system data to identify that patients hospitalised for more than 24 hours reported poorer health status at 12 months follow up in comparison to those not hospitalised after their accident. After adjustments for known risk factors such as age, injury severity and gender, multivariate analysis revealed no differences in health status between the two groups at 24 months [38].

#### 3.3. Procedural justice and compensation process

A number of quantitative studies have found an association between claiming compensation and poor recovery [32, 34, 45, 46]. However, it is unclear which aspect of the compensation system negatively impacts patients’ recovery. A quantitative study investigating health outcomes of people who sustained injuries in transport-related accidents found that those who were found not to be at fault for the accident experienced more emotional and mental disturbances than those who were at fault [46]. It has also been shown that people seeking legal involvement 12 months post-accident were more likely to be socio-economically disadvantaged pre-accident than those not seeking legal assistance [32], and that claiming compensation was associated with longer time to resolution of symptoms in patients with whiplash (30). Furthermore, interactions with insurance companies were found to be less fair than interactions with lawyers [34]. This might be due to the patients’ perception of seeing lawyers as allies, where insurance companies might have given them the feeling of being mistrusted.

In particular, one qualitative study explored this issue in more depth and reported that compensation patients seem to seek legal assistance after experiencing frustration with administrative requirements and claims procedures or when experiencing abandonment from the compensation system or lack of assistance. It has been suggested that pursuing legal assistance might be related to the complexities involved in claiming compensation and perceived poor-recovery, especially if patients feel that reasons for poor recovery were caused by being involved in compensation process. The study reported that patients perceived the claims processes too complex and supplemented by delays in receiving treatment approvals, which, in their opinion, adversely affected recovery and quality of care [56].

#### 3.4. Importance of terminology and timely healthcare decision making

The terminology of minor injury used in legislation and guidelines was identified by study participants as an issue in one study [57]. The study also reported that patients perceived the role of social insurers and healthcare practitioners as very important in the recovery process. The study emphasised the importance of developing a strong relationship between the patient and health care provider, which could be improved through the health care provider offering explanations, choices, and anticipatory teaching about treatment options in a manner that can be easily understood by patients.

The role of health professionals in timely decision making about treatment was also perceived as important, particularly for compensable treatments that needed to be approved by the social insurers. Lack of communication and systematic decision making between health and social professionals was identified as a problem and perceived as leading to poor quality of care and lack of care coordination.

#### 3.5. Family and social support

Social support was found to be an important factor in patients’ recovery. A cross-sectional study revealed that poor social support was associated with worse long-term functioning following whiplash injury. Everyday emotional support, emotional support during problems, appreciative support and informative support have shown a relationship with long-term functionality [49]. However, it was suggested that to investigate predictive capacity of this factor, prospective studies are necessary as the nature of this study was not able to draw a causal relationship with poorer recovery outcomes. On the other hand, one qualitative study has demonstrated and explained that family and social support was associated with people feeling safe and protected, yet, that might not always be the case as in presence of pain and disability, support does not necessarily lead to better recovery outcomes [56].

## Discussion

This review attempted to evaluate biological, psychological and social factors impacting recovery and to understand the inter-relationship between these factors and their representativeness across individual studies. Results of the 35 quantitative and 2 qualitative studies evaluated in this narrative review indicated that a range of multidimensional factors affect the recovery outcome of patients. The most stable and reliable finding was the relationship between high levels of pain and physical and mental disability. High initial levels of pain have shown to have a strong prognostic capacity immediately after the accident. It seems that early identification of intensity, localisation and duration of pain is essential in identifying high-risk groups and predicting recovery outcomes. The results are consistent with preceding reviews reporting a relationship between high initial levels of pain and chronic pain [13, 60]. Equally, pre-accident pain was also shown to play an important role in recovery prediction, as it is highly likely that if patients suffer pain prior to an accident, it will be exacerbated after the accident or progress into chronic pain. The substantial psychopathology has also been identified as many patients would suffer depression, PTSD and anxiety, especially if they have had previous mental health issues.

Other common factors associated with poor or non-recovery were older age, female gender, pain catastrophising, poor recovery expectation, pre-accident health status, previous unemployment, low educational level and work incapacity.

However, it is to note that Walton et al’s systematic review published in 2009 [60] contradicted an earlier review by Cote et al in 2001 [13] in finding that age was not associated with an outcome after whiplash injury. This review found that older people are prompt to suffer poorer recovery outcomes due to their previous chronic health conditions. Although age should not be considered as a definitive and direct indicator of recovery, this review shows that it is part of the cluster of factors associated with non-recovery and needs to be taken into account when determining high-risk groups.

Further, recovery beliefs and poor expectations have been shown to be associated with non-recovery [27, 61] with studies suggesting that intervening early in the recovery process and providing psychological support to at-risk people may facilitate better outcomes. Prompt assessment of the patient’s psychological status may assist in directing at-risk patients to psychological resources to improve their recovery expectations and beliefs, with an aim of improving their outcomes in general. Further research is required to understand what causes these negative beliefs, how those can best be screened and what strategies are most effective in improving patients’ expectations following physical and mental disability. This gap could likely be fulfilled with educating health professionals and rehabilitation coordinators and getting them involved to facilitate the recovery belief amongst patients. Hence, education on both sides has a key role in raising awareness among health professionals, insurers and patients of both physical and physiological injuries after transport accidents [28].

Understanding patients’ beliefs and experiences of pain and injury seems to be essential for pain management and prediction of recovery outcomes. In addition, these factors define patients’ personality and in combination with other aforementioned factors, give promising predictive capacity that is important for healthcare professionals as well as for social insurers. The review papers also suggest that, like pain and age, negative patients’ beliefs and poor coping skills are part of the cluster of factors associated with non-recovery and healthcare professionals and insurers should be aware of patients’ needs and common comorbidities after transport accidents [28].

Social factors such as lower educational level, family support, hospitalisation status, previous unemployment and compensation and legal involvement should be assessed cautiously and rigorously with other considerations taken into account, such as pain, depression, anxiety, return to work and ability to return to usual daily activities. There is notable evidence around the impact of compensation expectations on health outcomes or legal involvement being associated with non-recovery [36, 45, 46], yet further research is needed to explain these relationships, as the influence of these factors is usually not direct, but is affected with other confounding factors. Understanding factors that put patients into risk of non-recovery is a vital step into planning and organising their rehabilitation plan and therefore, patients should be assessed based on their individual circumstances, taking into account the aforementioned factors as potential obstacles in their recovery. Thus, recovery after a minor injury is not a one-way process, yet it involves a multi-faceted management and coordination.

This review has several strengths including comprehensive search strategy and an in-depth methodological quality assessment of individual studies. A large number of studies were assessed and the rigorous methodology used gives a high level of evidence of studies that investigated minor transport-related injuries and reported prognostic associations between factors and outcomes. The majority of papers included are of a prospective study design, which is considered to be an optimal research design to identify the existence of prognostic factors and their relationship with outcomes. However certain limitations exist and have to be noted. Firstly, data regarding the most prognostic factors associated with poor recovery was difficult to interpret due to heterogeneity of the techniques and tools used to assess such associations and the way in which they were reported. This prevented us from evaluating the relative importance of each risk factor on recovery.

Secondly, potential reference bias needs to be noted as screening references of identified studies may result in an over representation of negative studies in the review.

Thirdly, the use of univariate results, when multivariate results were not available, could have biased our conclusions regarding the level of evidence for a prognostic factor, because univariate results were not adjusted for potential confounding factors.

Finally, we report on risk of bias in the study design but have not considered the impact of this bias on each individual risk factor being examined.

In summary, understanding and preventing incapacity after minor injuries will require the development of guidelines and information protocols that addresses the physical, psychological and social factors involved in patients’ injury and disability.

The use of consistent tool to measure recovery outcomes should be a priority to improve interpretability and comparability of future studies. Even though previous reviews highlight the need of using consistent measure of recovery, this review demonstrates this is still not the case. The results are concerning as it was impossible to consolidate the literature even though the number of prospective studies with a long follow up have increased.

It may be beneficial to consider developing a recovery specific Patients Reported Outcomes Measure (PROMs) in order to enhance interpretability and consistency which could also improve screening process for high risk groups.

Further, health professionals should be aware that even though minor in nature, injuries may trigger pre-existing patient vulnerabilities, which may then lead to development of chronic disability. It is vital when assessing patients after an injury to look beyond the physical injury and whether the type of injury is considered minor, moderate or severe and consider other factors in this review which we now know will impact recovery. These principles are fundamental in achieving better recovery outcomes from clinical and rehabilitation management.

## Supporting information

S1 File Final search strategy. (PDF)

S2 File PRISMA checklist. (PDF)
