# Supplementary material for: Biopsychosocial factors associated with non-recovery after a minor transport-related injury: A systematic review
Source: PLoS One. 2018 Jun 12;13(6):e0198352. doi: 10.1371/journal.pone.0198352 (PMC5997362; doi:10.1371/journal.pone.0198352)
Supplement: S1 File — (PDF) [file pone.0198352.s001.pdf]

Final search MEDLINE

Concept A

Minor injuries (Musculoskeletal and soft tissue)

# ▲	Searches	Results	Type	Actions	Annotations
1	arm injuries/ or forearm injuries/ or wrist injuries/ or back injuries/ or fractures, cartilage/ or hand injuries/ or finger injuries/ or lacerations/ or leg injuries/ or ankle injuries/ or foot injuries/ or knee injuries/ or neck injuries/ or whiplash injuries/ or soft tissue injuries/ or "sprains and strains"/ or tendon injuries/ or contusions/ or head injuries, closed/ or brain concussion/	102856	Advanced	Display Results More ▾	
2	exp Musculoskeletal System/in [Injuries]	101016	Advanced	Display Results More ▾	
3	exp Whiplash Injuries/	3136	Advanced	Display Results More ▾	
4	((head* or neck* or shoulder* or arm* or forearm* or wrist* or hand* or finger* or upper limb* or upper extremit* or back* or pelvis* or pelvic* or leg* or knee* or foot* or ankle* or feet* or lower limb* or lower extremit* or toe*) adj3 (injur* or contusion or concussion* or abrasion* or laceration* or sprain* or strain*)).mp. [mp=title, abstract, original title, name of substance word, subject heading word, keyword heading word, protocol supplementary concept word, rare disease supplementary concept word, unique identifier]	120673	Advanced	Display Results More ▾	
5	whiplash*.mp.	3531	Advanced	Display Results More ▾	
6	(minor adj (injur* or contusion or concussion* or abrasion* or laceration* or sprain* or strain*)).mp.	1796	Advanced	Display Results More ▾	
7	1 or 2 or 3 or 4 or 5 or 6	220258	Advanced	Display Results More ▾	

Contract

Concept B

Transport-related accident/injury

#	Searches	Results	Type	Actions	Annotations
8	Accidents, Traffic/	40111	Advanced	Display Results More ▾	
9	((car or cars or truck or trucks or automobile* or cyclist* or cycling* or cycle* or pedestrian* or passenger* or driver* or motor* or vehicle* or vehicul* or transport* or traffic*) adj3 (accident* or collision* or crash* or smash*)).mp.	49562	Advanced	Display Results More ▾	
10	8 or 9	49562	Advanced	Display Results More ▾	

Concept C

Types of studies including limitations to English language and year 2000 – current

[-] Clinical Studies as Topic	[illegible]
[-] ☑ Epidemiologic Studies	7951
[-] ☑ Case-Control Studies	250992
☑ Retrospective Studies	643251
[-] ☑ Cohort Studies	233587
☑ Follow-Up Studies	595124
[-] ☑ Longitudinal Studies	120932
☐ National Longitudinal Study of Adolescent Health	125
☑ Prospective Studies	464937
☑ Retrospective Studies	643251
☐ Controlled Before-After Studies	208
☑ Cross-Sectional Studies	255004
☐ Historically Controlled Study	87

11	7 and 10	10964	Advanced	Display Results More
12	epidemiologic studies/ or case-control studies/ or retrospective studies/ or cohort studies/ or follow-up studies/ or longitudinal studies/ or prospective studies/ or cross-sectional studies/	2109829	Advanced	Display Results More
13	qualitative research/	33787	Advanced	Display Results More
14	case control.mp.	277453	Advanced	Display Results More
15	((follow up or followup) adj (study or studies)).mp.	612199	Advanced	Display Results More
16	(observational adj (study or studies)).mp.	82240	Advanced	Display Results More
17	((observational or prospective or retrospective) adj (study or studies)).mp.	1191717	Advanced	Display Results More
18	Cross sectional.mp.	324661	Advanced	Display Results More
19	(cohort adj (study or studies)).mp.	296992	Advanced	Display Results More
20	(qualitative adj (study or studies)).mp.	23731	Advanced	Display Results More
21	12 or 13 or 14 or 15 or 16 or 17 or 18 or 19 or 20	2331533	Advanced	Display Results More
22	11 and 21	3108	Advanced	Display Results More
23	limit 22 to (english language and humans and yr="2006 -Current")	1401	Advanced	Display Results More
